# MCL1 inhibition targets Myeloid Derived Suppressors Cells, promotes antitumor immunity and enhances the efficacy of immune checkpoint blockade

**DOI:** 10.1038/s41419-024-06524-w

**Published:** 2024-03-08

**Authors:** Nabanita Mukherjee, Elizabeth Katsnelson, Tonya M. Brunetti, Kylie Michel, Kasey L. Couts, Karoline A. Lambert, William A. Robinson, Martin D. McCarter, David A. Norris, Richard P. Tobin, Yiqun G. Shellman

**Affiliations:** 1 University of Colorado Anschutz Medical Campus, School of Medicine, Department of Dermatology, Aurora, CO 80045 USA; 2 University of Colorado Anschutz Medical Campus, School of Medicine, Division of Surgical Oncology, Aurora, CO 80045 USA; 3 Department of Immunology & Microbiology, University of Colorado Anschutz Medical Campus, Aurora, CO USA; 4 University of Colorado Cancer Center, University of Colorado Anschutz Medical Campus, Aurora, CO USA; 5 University of Colorado Anschutz Medical Campus, School of Medicine, Division of Medical Oncology, Aurora, CO 80045 USA; 6 Department of Veterans Affairs Medical Center, Dermatology Section, Denver, CO 80220 USA; 7 University of Colorado Anschutz Medical Campus, Gates Center for Regenerative Medicine, Aurora, CO 80045 USA

**Keywords:** Cancer therapeutic resistance, Apoptosis

## Abstract

Immune checkpoint inhibitors (ICIs) are now the first-line treatment for patients with advanced melanoma. Despite promising clinical results, many patients fail to respond to these therapies. BH3 mimetics, a novel class of small molecule inhibitors that bind and inhibit anti-apoptotic members of the BCL2 family proteins such as BCL2 or MCL1, have been very successful in treating hematologic malignancies. However, there are limited studies on the immunomodulatory role of the BH3 mimetics. Several factors contribute to ICI resistance including myeloid-derived suppressor cells (MDSCs) that exert immunosuppressive effects through direct and indirect inhibition of antitumor immunity. Thus, targeting MDSCs to enhance antitumor immunity has the potential to enhance the efficacy of ICIs. In this study, we show that the MCL1 inhibitor S64315 reduces melanoma tumor growth in an immune cell-dependent manner in mice. Specifically, S64315 enhances antitumor immunity by reducing MDSC frequency and by promoting the activity of CD8+T cells. Additionally, human MDSCs are 10 times more sensitive to S64315 than cutaneous melanoma lines. Further, we found that a higher expression of MCL1 is associated with poor survival for patients treated with anti-PD-1. Finally, combining S64315 and anti-PD-1 significantly slowed tumor growth compared to either agent alone. Together, this proof-of-concept study demonstrates the potential of combining an MCL1 inhibitor with anti-PD-1 in the treatment of melanoma. It justifies the further development of next generation MCL1 inhibitors to improve efficacy of ICIs in treating malignant melanoma.

## Introduction

Melanoma is a deadly form of cancer arising from melanocytes and remains a significant and rising health burden in the United States. Despite significant advances in the development of immune checkpoint inhibitors (ICIs) and their improvement of clinical outcomes for melanoma patients, tumors in a significant proportion of patients are or will become resistant to these revolutionary therapies. Therefore, there is an urgent need to find new therapies to improve the efficacy of current treatments for melanoma. The resistance of melanoma to many ICIs is driven by a variety of often overlapping mechanisms [1–4]. In particular, Myeloid-derived suppressor cells (MDSCs) pose a significant obstacle to successful antitumor immunity and effective ICI treatment by leveraging many of these resistance mechanisms [5–7]. This study aims to explore the therapeutic potential of MCL1 inhibitors to target MDSCs and improve the efficacy of ICIs for melanoma.

MCL1 is an anti-apoptotic member of the BCL2 family, which is the primary regulatory protein controlling the intrinsic apoptosis pathway [8–11]. This family includes both pro-apoptotic and anti-apoptotic proteins, which share similarities in the BCL2 homology (BH) domains and act primarily through the regulation of caspases or mitochondrial apoptogenic factors [9, 10, 12, 13]. Dysregulation of the BCL2 family proteins occurs in many cancers and contributes to the tumorigenesis and development of acquired resistance to targeted or chemotherapies [14–18]. This has led to the development of a new class of drugs called BH3 mimetics, which are small molecule inhibitors that mimic the action of pro-apoptotic family members and selectively inhibit the anti-apoptotic members. One of the most successful BH3 mimetics, venetoclax, a selective BCL2 inhibitor, has proven to be highly effective in the treatment of patients with hematologic malignancies [19, 20].

The promising results from venetoclax have prompted the exploration of other BH3 mimetics, including MCL1 inhibitors. Many solid cancers upregulate MCL1, and melanoma in particular is dependent on MCL1 for survival [21–23]. Elevated levels of MCL1 expression in melanoma patient tumors have been linked with worse responses to targeted or chemotherapies [21, 24]. In recent years, there has been a growing interest in the use of MCL1 inhibitors to treat solid tumors including melanoma [21, 25–28]. However, to date, none of these prior studies have examined the immunomodulatory properties of the MCL1 inhibitor S64315, which is the focus of this study.

MDSCs are a significant source of treatment resistance in various cancers including melanoma [29–31]. MDSCs are a heterogeneous population of immature myeloid cells that accumulate in cancer. The presence of these cells in both the tumor and the periphery promotes tumor growth and immune escapees through the production of immunosuppressive molecules such as interleukin-10 (IL-10), reactive oxygen species (ROS), vascular endothelial growth factor (VEGF), and the expression of cell surface receptors that suppress T cell responses [32, 33]. ICIs rely on proper immune function to be effective and the dampening of T-cell responses by immunosuppressive populations such as MDSCs has been shown to be a factor in the low response rates to therapy [29–31, 34, 35].

There are two primary subsets of MDSCs in humans, CD15^+^CD14^-^ polymorphonuclear MDSCs (PMN-MDSCs) and CD15^-^CD14^+^ Monocytic (MO-MDSCs) [36]. PMN-MDSCs are defined as Ly6G^-^Ly6C^high^ and MO-MDSCs as Ly6G^-^Ly6C^low^ [36, 37]. In metastatic melanoma patients, an increased frequency of circulating MDSCs has been associated with decreased responses to ICIs due to their immunosuppressive effects on the tumor microenvironment [38]. Importantly, MCL1 has been shown to be a critical factor in the survival and development of MDSCs [39]. Therefore, we hypothesized that the MCL1 inhibitor S64315 will decrease MDSC frequency and promote effective antitumor immunity.

This study investigates the therapeutic potential of S64315 as an immunomodulatory agent, not only in mouse melanoma models, but also in human immune cells. Using mouse models, we determined the effects of the MCL1 inhibitor S64315 on tumor growth, tumor-infiltrating MDSC frequency, CD8^+^ T cell function, and the efficacy of anti-PD-1 therapy. Using human cells, we examined the effects of S64315 on MDSCs and T cells. Overall, our findings emphasize the need for the development and testing of next-generation MCL1 inhibitors in combination with ICI therapy to improve outcomes for patients with metastatic melanoma.

## Materials and methods

### Cell lines, reagents, and drug treatments

Mouse (B16.F10 and YUMM 1.7) and human (A375) melanoma cell lines (ATCC, Manassas, VA) were used for in vitro and in vivo studies. YUMM1.7 and A375 are BRAF mutated and B16.F10 is BRAF WT.

The MCL1 inhibitor (S64315/MIK-665) was purchased from MedChem Express (Monmouth Junction, NJ, USA). Anti-PD-1 (Catalog #BE0273) and the isotype control (Catalog #BE0089) were purchased from Bio X Cell (Lebanon, NH, USA). Details of other antibodies are provided in Supplementary Table 1.

### In vitro studies

The melanoma cell lines B16.F10 and A375 were cultured in RPMI with 10% FBS (Gemini Bio-Products, Inc., West Sacramento, CA, USA), while YUMM 1.7 was cultured in DMEM: F12 with 10% FBS. HL-60 cell line was obtained from the Cell Technologies Shared Resource University of Colorado, Anschutz Medical Campus. HL-60 was cultured in Iscove’s Modified Dulbecco’s Medium (IIMDM, GlutaMAX™ Supplement) (Gibco; 31980-030) with 20% FBS.

For viability assays, the cells were seeded at the density of 4,000 viable cells in 100ul in 96-well plates. The cells were treated with the vehicle control or various concentrations of S64315 on the next day. The viability assay was conducted at 24 h post treatment using Cell Titer-Glo Luminescent cell viability kit (Promega Corp., Madison, WI, USA), similarly as in [23, 26–28, 40].

The MCL1 knockdown lines were created using B16.F10 and YUMM 1.7 cell lines, similarly as previously described [23, 26–28, 41–43]. Specifically, the cells were treated with sh Control (sc-108080) or sh MCL1 (sc-35878-V) lentiviral particles along with Polybrene (sc-134220; final concentration of 5 µg/ml) overnight. All reagents were purchased from Santa Cruz Biotechnology (Dallas, TX, USA). The cells were then allowed to recover from the transduction process for 24 h. The cells were then subjected to puromycin selection for 14 days at a final concentration of 2 μg/mL. The knockdown level was tested by immunoblot using MCL1 (sc-74436) and Tubulin (#2148) antibodies. The tubulin antibody was purchased from Cell Signaling Technologies (Danvers, MA, USA).

### Mouse studies

**Fig. 1:**
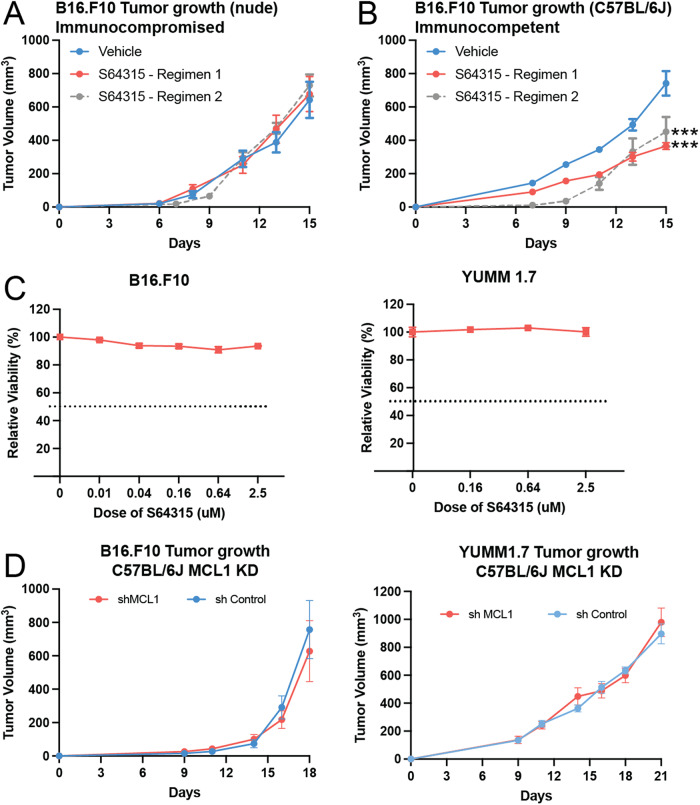
The MCL1 inhibitor S64315 only functions in immunocompetent hosts. Effects of the MCL1 inhibitor S64315 on growth of B16.F10 tumors in immunocompromised nude mice (**A**) or in immunocompetent C57BL/6 J mice (**B**). Day 0 was the day of cell implantation. Treatments were 25 mg/kg, at the Regimen 1 (Days 4, 6, 11, and 13) or Regimen 2 (Days 4, 6, 9, 11, and 13). **C** Effects of S64315 on B16.F10 and YUMM1.7 cells in vitro. Y-axis shows the relative viability compared to the cells treated with the vehicle control, and dashed line indicates 50% viability. **D** Effects of MCL1 knockdown of B16.F10 and YUMM1.7 on their tumor growth, with cells of the control (sh Control) and knockdown (sh MCL1). ***p < 0.001. Error bars represent ±SEM for all figures. Immunoblot of the knockdown are in Supplementary Fig. 1.

All animal experiments were approved by the Institutional Animal Care and Use Committee (IACUC) of the University of Colorado Denver (protocol #318) and were conducted using 8–10-weeks-old female C57BL6J mice or NCRNU nude mice. For all mice experiments, mice were implanted with 100ul of cell suspension containing 200,000 cells (for B16.F10) or 100,000 cells (for YUMM1.7) in PBS or in a suspension of 50% Matrigel and media (Fig. 5C vs Fig. 1A, B). All studies had 5 mice per treatment group. The number of mice per group was chosen based on literature search and calculations using PASS statistical software. S64315 was used at a dose of 25 mg/kg and was administered through intraperitoneal route unless otherwise mentioned. S64315 was prepared by dissolving the drug in 2% kolliphor and 98% sterile PBS. Kolliphor EL, formerly known as Cremophor EL was purchased from EMD Millipore (Cat No. 238470). For flow cytometry studies, S64315 was administered on days 4, 6, and 9, and the tumors were harvested on day 10. All in vivo treatment begins 4 days post tumor inoculation. For Fig. 1B, 10-week-old mice were used, and the vehicle was the control for S64315 alone. For Fig. 5C, 8-week-old mice were used, and the vehicle controls included those for both S64315 and Anti-PD-1.

For the combination treatments of the S64315 plus anti-PD-1, the mice were randomly divided into four groups. Anti-PD-1 was administered at the dose of 10 mg/kg on days 5, 7, and 10; S64315 was administered at the dose of 25 mg/kg on days 4, 6, 9, and 11.

For mouse studies with MCL1 knock down lines, B16.F10 and YUMM1.7 sh Control or sh MCL1 stable cell lines were created using shRNA lentiviral particles (Santa Cruz Biotechnology, Dallas, TX, USA). Immunoblot was used to confirm the knockdown level.

### Melanoma patient samples

Tumor samples from melanoma patients were collected from the University of Colorado Hospital under the Melanoma Biorepository at the University of Colorado Cancer Center COMIRB #05-0309. Tumors were first mechanically dissociated into pieces < 1mm^3^ and then chemically digested at 37 °C for 30 minutes with 5 µg mL−1 Liberase DL (Roche). Following enzymatic digestion, tumors were passed through a 40 μm cell strainer to create a single-cell suspension.

### Healthy donor samples

Peripheral blood was collected from healthy donors from the University of Colorado Anschutz Medical Campus Clinical and Translational Sciences Institute COMIRB protocol #17-2159. Blood samples were collected in yellow-top ACD tubes and PBMCs were isolated by gradient centrifugation with Ficoll-Paque PREMIUM (1.077 g/mL) sterile solution.

All patient studies were conducted in accordance with the Declaration of Helsinki, Belmont Report, and U.S Common Rule. All patients were consented under approval from the Colorado Institutional Review Board (COMIRB #05-0309 and #17-2159) and provided written consent for the utilization of samples for data collection and publication. Details of the patients sample provided in Supplementary Fig. 2B.

### Single-cell RNA-seq

Myeloid cells were isolated from two separate melanoma tumors by fluorescence-activated cell sorting. Live CD45+/CD3-/CD19-/CD56-/CD11b+ (antibody information can be found in Supplementary Table 1) myeloid cells were sorted on the Sony MA900 cell sorter. Single cells were captured on the 10X Genomics Chromium system and libraries were prepared for sequencing. Libraries were sequenced using an Illumina NovaSeq at University of Colorado Cancer Center Genomics Shared Resource.

### Single-cell RNA-seq analysis

Quality control and read mapping were performed using the 10X Genomics Cell Ranger analysis pipeline on the 10X Genomics Cloud Analysis Server. Count and meta data were loaded into Seurat (v4.3.0) for quality filtering of cells based on mitochondrial contamination ( < 20% of unique molecular identifier counts), unique genes identified (nFeature_RNA > 300 per cell), total molecules detected (nCount_RNA > 500 per cell), and cell complexity (>0.80). Normalization, clustering, and dimension reduction were performed in Seurat. The sample from one patient failed filtering and quality control. Our analysis was focused on myeloid cells, and any contaminating cells expressing melanoma genes (MLANA, MITF, and TYR) were filtered from the analysis. All code used in this study is available at https://github.com/UCHRT/Tobin_Lab.

### MDSC differentiation from human monocytes

All research subjects provided written informed consent under the COMIRB-approved protocol #17-2159. Monocytes were isolated from PBMCs using the MojoSort Human CD14 Selection Kit (BioLegend), and incubated for 7 days in10ng/ml GMCSF,10ng/ml IL-6, and RPMI supplemented with 1% l-Glutamine, 1% Penicillin/Streptomycin, 1% sodium Pyruvate, 1% 1 M Hepes, and 55μM 2-Mercaptoethanol. Medium was refreshed on day 4 (as previously described) [31].

### Human T cell activation

Total PBMCs from the healthy donor samples described above were activated with anti-CD3/anti-CD28 microbeads (Dynabeads, Thermo Fisher) for 48 h according to the manufacturer’s protocol, in the presence of a dose-response of S64315 or DMSO vehicle. After 48 h, the beads were magnetically removed from the culture media, and the cells were incubated in the presence of S64315 or DMSO until day 7. On day 7, the cells were moved to a 96-well round bottom plate coated in anti-CD3 and incubated in the presence of monensin (2μM, Biolegend) for 6 h. T-cell activation was then measured by intracellular cytokine staining.

### Flow cytometric analysis

Cells from tumors and PBMCs were stained in flow staining buffer containing 5% BSA and 0.05 M EDTA for all extracellular staining. A full list of flow cytometric antibodies can be found in Supplementary Table 1. The ebioscience FoxP3/Transcription Factor Staining Buffer Set (eBioscience) was used for all intracellular staining. Fc blocking was done at 4 °C for 20 minutes with either 2.4G2 for the mouse samples or heat inactivated human serum (HIHS) for the human samples. All cells were stained with Live/Dead discrimination Ghost Dye 780 (13-0865-T100, Tonbo Biosciences). A complete list of the antibodies used is included in the supplement. Flow cytometric analysis was performed on a Beckman Coulter CytoFlex S flow cytometer, and data was analyzed using FlowJo v10 Software (BD Biosciences).

### Statistical analysis

KM plot for MCL1 was created from the transcriptomic datasets of cancer patients treated with Anti-PD-1. We used https://kmplot.com/analysis/index.php?p=service&cancer=immunotherapy to create the KM plot, and selected all tumor types, which include bladder, esophageal, glioblastoma, hepatocellular carcinoma, HNSCC, melanoma, NSCLC, NSLC, and urothelial cancer. For the KM plot statistics, we set the significance criteria of p-value less than 0.001 and FDR less than 5%. Website: Kaplan–Meier plotter [Immunotherapy] (kmplot.com) [44]. Date accessed: 1/27/2023. In the search criteria, we included all tumor types and included patients treated with any anti-PD-1 drugs, Nivolumab or Pembrolizumab.

Graphs for Figs. 1, 4A, and 5C were created using GraphPad Prism V8 software, and statistical analyses were conducted using GraphPad Prism V8 software. The distribution of data was determined using the included D’Agostino-Pearson test for skewness and kurtosis. When comparing differences between two groups, two-tailed unpaired t-tests were utilized to determine statistical significance. IC50 values were calculated from the relative viability results of ATP assays and were derived by a sigmoidal dose (log)-response (variable slope) curve with GraphPad Prism 8 software. We used two-way ANOVAs, followed by recommended post-hoc tests, to evaluate if the experimental groups were significantly different. Data from combination studies were analyzed by two-way/mixed model ANOVA, followed by recommended post-hoc tests. For all of these analyses, p-value less than 0.05 was considered significant.

## Results

### The MCL1 inhibitor S64315 has an antitumor activity in immunocompetent mice but not immunocompromised mice, indicating its immunomodulatory effect

To establish the preclinical therapeutic potential of the MCL1 inhibitor S64315 in melanoma, we began our investigation by comparing the efficacy of this agent in both immunocompromised nude mice, which lack functional lymphocyte populations [45], and immunocompetent C57BL/6 J mice. While we have previously shown that MCL1 inhibitors can have direct tumor killing activity in some human uveal melanoma cell lines [27], we did not observe any statistically significant change in the growth of B16.F10 melanoma tumors in the immunocompromised nude mice (Fig. 1A). However, tumor growth was significantly reduced by S64315 in the immunocompetent C57BL/6 J mice (Fig. 1B, p < 0.001), suggesting an immunomodulatory role for this MCL1 inhibitor. We tested two different treatment regimens (Regimen 1: days 4, 6, 11 and 13; Regimen 2: days 4, 6, 9, 11 and 13). In the nudes, neither regimen had any effect on the tumor growth (Fig. 1A); while in the C57BL6J mice, both regimens inhibited tumor growth and there was no significant difference in the tumor response between the two regimens (Fig. 1B). Overall, these results indicate that this MCL1 inhibitor requires immune cells for its antitumor activity. We further examined the direct effects of inhibiting MCL1 specifically in tumor cells, in vitro and in vivo, using both B16.F10 and YUMM 1.7 cells (Fig. 1C, D). In vitro experiments using these cell lines showed that S64315 had little to no direct effect on cell viability (Fig. 1C). In vivo, we performed shRNA knockdown of MCL1 in these cell lines prior to implantation into C57BL/6 mice (Fig. 1D). MCL1 knockdown in tumor cells (confirmed in Supplementary Fig. 1) was also not sufficient to inhibit tumor growth in immunocompetent mice, since there was no difference in tumor growth between control and MCL1 knockdown cells with either cell line (Fig. 1D). Collectively, these results indicate that S64315 is functioning in vivo through the modulation of immune cells.

### MDSCs from anti-PD-1 resistant human melanoma tumor express high levels of MCL1

**Fig. 2:**
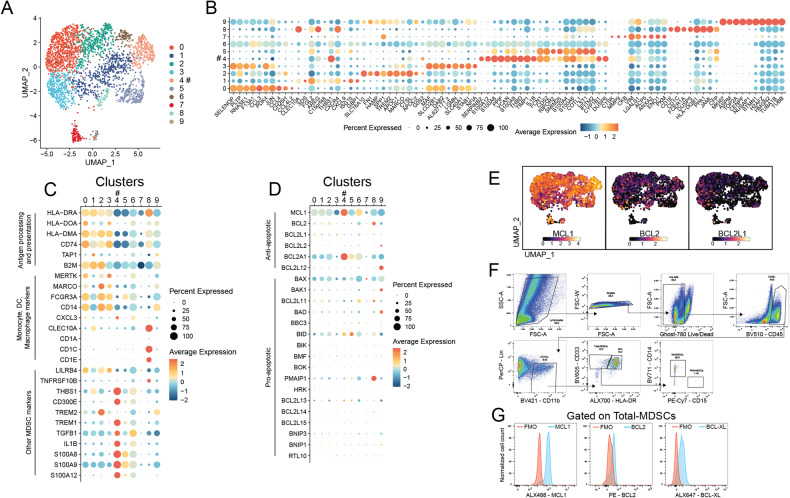
MDSCs from anti-PD-1 resistant human melanoma tumor express high levels of MCL1. **A** UMAP projection of live/CD45+/CD3-/CD19-/CD56-/CD11b+ cells from one melanoma tumor (omental metastasis from 30-year-old patient that experienced progressive disease following 17 cycles of pembrolizumab). **B** Dot plot depicting the top 10 unique genes that define each of the clusters. **C** Dot plot depicting genes associated with the MDSC transcriptional profile. **D** Dot plot depicting the expression of genes in the BCL2 family. **E** Feature plots showing the distribution of the relative expression of MCL1, BCL2, and BCL2L1 respectively, as clustered in **A**. **F** Representative flow cytometric gating strategy for the analysis of MCL1, BCL2, and BCLXL expression in MDSCs from melanoma patients. **G** Representative histogram of flow cytometric analysis of MCL1, BCL2, and BCLXL expression induced by the labeled cytokines in MDSCs, gated as shown in **F**. Data in **G** are representative graphs from 10 separate melanoma patients (Supplementary Fig. 2), comparing the labeled fluorochrome conjugated antibody to the corresponding fluorescence minus one (FMO) staining control. Y-axis values are normalized to the cell count in each sample. X-axis label includes antibody specificity and fluorochrome conjugate. # indicates cells expressing the MDSC transcriptional profile.

Previous reports have found MCL1 as a critical factor in the survival and development of MDSCs [39]. Therefore, we sought to determine the expression of MCL1 across myeloid cell populations in tumors from patients relapsed from anti-PD-1 treatment. We performed single-cell RNA-sequencing (scRNAseq) on fluorescence-activated cell sorting purified myeloid cells (Fig. 2A). As has been previously described [46–48], this analysis revealed heterogenous populations of tumor-infiltrating myeloid cells including multiple subsets of MARCO and MERTK expressing macrophages, CD14 and CD16 (FCGR3A) expressing monocytes, and dendritic cells (DCs) expressing CLEC10A, CD1C, and CD1E (Fig. 2B, C). Cluster 4 represents the myeloid cells expressing an MDSC-like phenotype, which includes low antigen processing and presentation machinery (HLA-DRA, CD74, HLA-DMA) and high expression of immunosuppressive genes (TGFB1, CD300E) (Fig. 2B, C) [6, 49]. These cells are separated from other tumor-infiltrating myeloid cells. Further gene expression analysis revealed that the MDSC-like cells in cluster 4 expressed high levels of MCL1, compared to other cells (Figs. 2D and 2E). In addition, this analysis indicated that MDSCs expressed high levels of BCL2A1, an additional and much less well described anti-apoptotic member of the BCL2 family (Fig. 2D). We have previously found that BCL2A1 is associated with decreased sensitivity to MCL1 inhibition in tumor cells [27]. Interestingly, this analysis also showed that the DC cluster (cluster 8) expressed high levels of BCL2 (Fig. 2D). This was not found amongst the other myeloid cell populations and BCL2 has recently been reported to control DC functions and to be associated with poor outcomes in anti-PD-1 treated patients [50].

We further confirmed the expression of MCL1 in MDSCs from other 10 melanoma tumors using flow cytometry (Fig. 2F, G and Supplementary Fig. 2A, B). As in the scRNA-seq analysis of the BCL2 family (Fig. 1D), tumor-infiltrating MDSCs expressed higher levels of MCL1 than the other major anti-apoptotic proteins BCL2 and BCL-XL (Fig. 2G). These results implicate the potential importance of MCL1 for MDSC survival or immunosuppressive functions.

### The MCL1 inhibitor S64315 decreases tumor-infiltrating MDSC frequency and increases intertumoral T cell function in C57BL/6 J mice

**Fig. 3:**
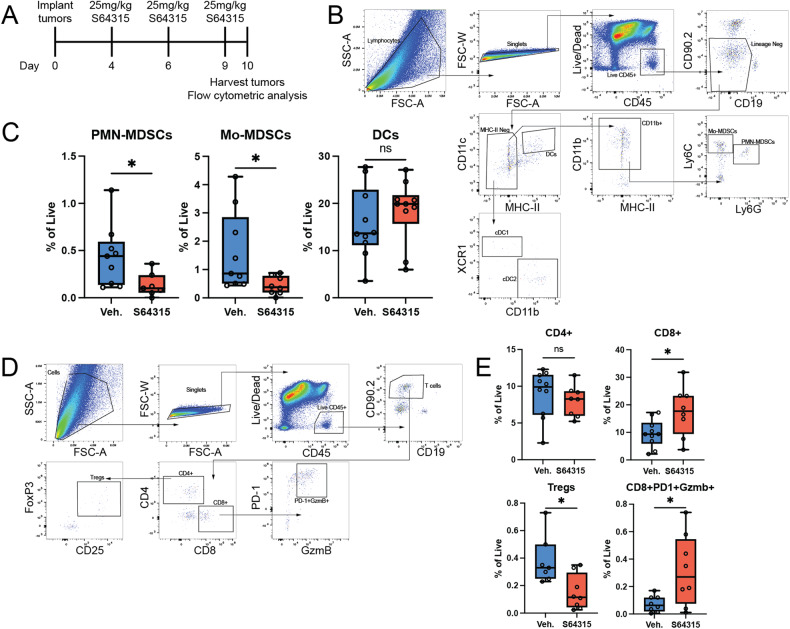
MCL1 inhibitor promotes antitumor immunity. **A** In vivo treatment schema. **B** Flow cytometric gating strategy for the analysis of tumor-infiltrating myeloid cells in C57BL/6 J mice treated as in **A**. **C** Comparisons of the frequency of PMN-MDSCs (Live, CD45+, CD19−, CD90.2−, MHCII−, CD11b+, Ly6C−, Ly6G+), MO-MDSCs (Live, CD45+, CD19−, CD90.2−, MHCII−, CD11b+, Ly6C+, Ly6G−) and dendritic cells (DCs, (Live, CD45+, CD19−, CD90.2−, CD11c+, MHCII+) as a percentage of the total live cells from each tumor. **D**. Flow cytometric gating strategy for the analysis of tumor-infiltrating T cells in C57BL/6J mice treated as in **A**. **E** Comparisons of the frequency of CD4+T cells, CD8+T cells, Tregs (CD4+, CD25+, FoxP3+), and activated PD-1+GzmB+ CD8+T cells. Five mice were treated per group with two tumors on each mouse analyzed. *p < 0.05. Data are representative of at least two separate experiments. See Supplementary Fig. 3 for gating strategy.

To further determine the immunomodulatory effects of S64315, we performed flow cytometric analysis of tumor-infiltrating immune cells from the tumors grown in the C57BL/6 J mice. These mice were implanted with B16.F10 tumor cells and treated with 25 mg/kg S64315, as shown in Fig. 3A. The gating strategy for mouse tumor studies is demonstrated in Supplementary Fig. 3. This dose of MCL1 inhibitor dramatically reduced the frequency of PMN- and MO-MDSCs (Figs. 3B and 3C). While there was a dramatic reduction in the frequency of both populations of MDSCs, there were no statistically significant differences in the frequency of dendritic cells (DCs) (Fig. 3C). The decrease in MDSCs was also associated with significantly decreased frequencies of Regulatory T cells (Tregs) and increased frequencies of total CD8+T cells and highly activated CD8+PD-1+Gzmb+ T cells (Figs. 3D, E). Overall, these data indicate that the antitumor immunomodulatory effects of S64315 are related to a significant reduction in immunosuppressive MDSCs.

### The MCL1 inhibitor S64315 can effectively kill human MDSCs in vitro

**Fig. 4:**
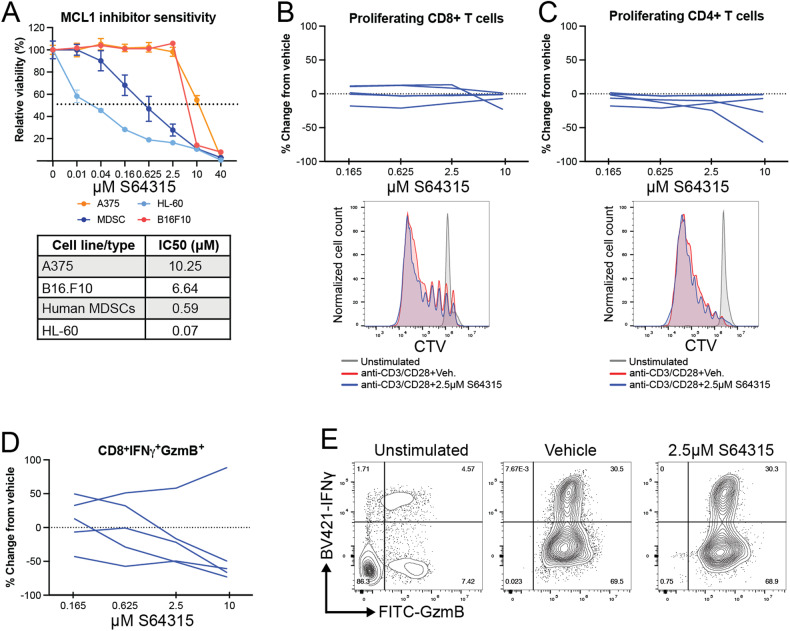
MCL1 inhibitor targets MDSCs and does not negatively impact T cell activation. Human MDSCs and T-cells were generated from five separate healthy donors. **A** Viability assays with human MDSCs, human melanoma cell line A375, mouse melanoma line B16.F10, and human MDSC-like cell line HL-60 after 24 h treatments with various doses of the MCL1 inhibitor S64315 in vitro. The bottom panel shows the IC50 values. **B** Quantification of the CD8+T cell proliferation in PBMCs activation with anti-CD3/CD28 beads analyzed at 7 days post activation. Example histogram of flow cytometric analysis of CD8+T cell proliferation. **C** Quantification of the CD4+T cell proliferation in PBMC activation with anti-CD3/CD28 beads, analyzed at 7 days post activation. Example histogram of flow cytometric analysis of CD4+T cell proliferation. **D** Quantification of intracellular cytokine staining for CD8+T cell expression of IFNγ and GZMB at 7 days post activation. **E** Representative contour plots showing the expression of IFNγ and GZMB in CD8+T cells. ***p < 0.001. *Y*-axis shows percentage of relative viability and *X*-axis indicates the dosages of the MCL1 inhibitor S64315 in µM. The dashed line indicates 50% viability. Error bars represent ±SEM for all figures.

To test the effects of the MCL1 inhibitor S64315 on the viability of human MDSCs, we conducted in vitro tests with MDSCs isolated from 5 healthy human subjects and an acute promyelocytic leukemia cell line HL-60 (an immature MDSC model) (Fig. 4A). HL-60 has high endogenous level of MCL1 [51]. For all these cultures, treatment with S64315 for 24 h resulted in a dose-dependent effect and significantly reduced the viability of both human MDSCs and the MDSC cell line HL-60; in contrast, it had a negligible effect on the viability of the human and mouse melanoma cell lines A375 and B16.F10 (Fig. 4A). These data indicate that this MCL1 inhibitor can kill human MDSCs at nM ranges and that MDSCs were ten times more sensitive to MCL1 inhibition than were the melanoma cell lines.

### MCL1 inhibition does not inhibit human T-cell proliferation or function

To further examine the therapeutic potential of MCL1 inhibitor in humans and how it may impact other immune cell subsets important for antitumor immunity, we performed in vitro T cell activation experiments with cells from the same 5 healthy donors as above. While the MCL1 inhibitor was able to effectively kill human MDSCs and MDSC-like cell lines at the nM dose range, no changes were observed in the proliferation of either human CD4+ or CD8+T cells at various doses above and below physiologically relevant concentrations of S64315 (Figs. 4B, C). Furthermore, S64315 did not alter the capacity of human CD8+T cells to produce IFNγ or Gzmb at 2.5 uM (a dose killing the majority of MDSCs) or lower (Figs. 4D, E). Although at the high doses, S64315 decreased this capacity in some samples, the reduction did not reach statistical significance. Additionally, these data indicate that the MCL1 inhibitor S64315 does not negatively impact T cell function, allowing them to maintain their normal functionality in the absence of MDSCs, and further illustrating the potential of this proof-of-concept experiment to promote effective antitumor immunity.

### High MCL1 expression is associated with poor survival in patients treated with anti-PD-1

**Fig. 5:**
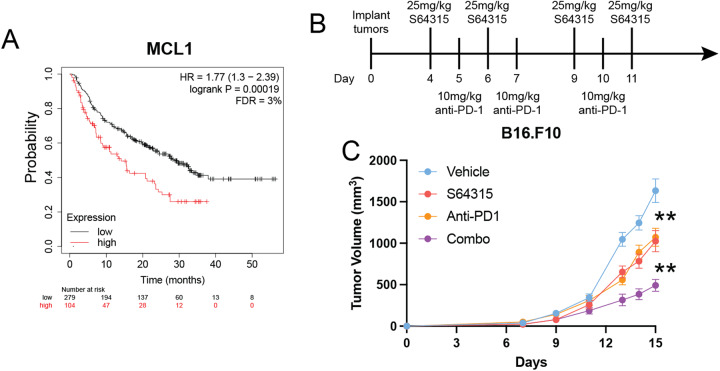
High MCL1 expression is associated with poor survival with patients treated with anti-PD-1 and MCL1 inhibitor enhances the potency of anti-PD-1 in vivo. **A** KM plot (kmplot.com) created from transcriptomic dataset of cancer patients treated with Anti-PD-1 based on the expression of MCL1. The *p*-value and FDR value are listed in the graph. **B** Combination treatment schema. **C** Tumor volumes in the C57BL/6J syngeneic mouse model with melanoma line B16.F10. The combination treatment of S64315 with anti-PD-1 significantly inhibited the tumor growth, compared to vehicle or the single drugs for multiple days. For visual clarification, we marked only the last day. **p < 0.01. Within each significant treatment, the least significant *p*-value of the comparisons is displayed. Error bars represent ±SEM for all figures.

To understand whether the expression of MCL1 in human tumors may affect response to anti-PD-1 treatment, we utilized the Kaplan–Meier Plotter at https://kmplot.com/analysis/, to visualize the relationships between the MCL1 expression and the overall survival of patients treated with anti-PD-1 (Fig. 5A). The Kaplan–Meier Plotter is a transcriptomic dataset of tumor samples with the correlated clinical data of cancer patients [44]. A lower p-value and false discovery rate (FDR) indicates that the significance of the association is strong. In these analyses, MCL1 was associated with significantly worse overall survival in anti-PD-1 treated patents compared to those with low expression (Fig. 5A). These data suggest that a higher expression of MCL1 decreases the probability of survival for the patients treated with anti-PD-1. We therefore hypothesize that inhibiting MCL1 will enhance the efficacy of the anti-PD-1 treatment.

### The MCL1 inhibitor S64315 enhances the efficacy of anti-PD-1 in a C57BL/6J syngeneic mouse melanoma model

We thus tested our hypothesis in the C57BL/6 J syngeneic model (Figs. 5B, C). Indeed, S64315 (25 mg/kg) in combination with anti-PD-1 (10 mg/kg) significantly inhibited B16.F10 tumor growth (*p* < 0.01), compared to the vehicle control or to a single agent (Fig. 5C). The combination was tolerable at these doses and did not significantly reduce mouse weight (Supplementary Fig. 4). Thus, the MCL1 inhibitor S64315 can enhance the efficacy of anti-PD-1.

## Discussion

Although ICIs have revolutionized cancer treatment, many melanoma patients do not respond to ICI therapy, making it necessary to explore alternative therapeutic approaches. In recent years, there has been an interest in exploiting the anti-apoptotic pathway by targeting the BCL2 family proteins to sensitize cancer cells to apoptosis and reduce the development of therapy resistance. While the feasibility of using MCL1 inhibitors to target melanoma has been shown [23, 26–28, 40–43], previous studies have not investigated the effects of the MCL1 inhibitor on immune cells.

This study is the first to report the antitumor immunomodulatory effects of the MCL1 inhibitor S64315 in melanoma. The use of multiple mouse models as well as primary human immune cells and cell lines makes this study a highly translationally relevant project to develop new melanoma treatments. These results provide evidence for a new mechanism to improve ICIs by targeting the pro-survival functions of MCL1 in myeloid cells, thus effectively decreasing the frequency of immunosuppressive MDSCs within the tumor microenvironment.

We hypothesized that the MCL1 inhibitor would decrease MDSC frequency and enhance the efficacy of ICIs. In vitro studies showed that human MDSCs were significantly more sensitive than melanoma cancer cell lines to the MCL1 inhibitor. Importantly, MCL1 inhibition did not impact human T cell proliferation or function, suggesting that the lower doses of S64315 have a greater specificity for MDSCs, thus making MCL1 inhibitors and anti-PD-1 therapy an attractive combination. In syngeneic immunocompetent mouse models, S64315 alone reduced the frequency of MDSCs resulting in improved CD8^+^ T cell function. The combination of this MCL1 inhibitor and anti-PD-1 therapy in these mouse models showed enhanced inhibition of melanoma growth and improved CD8^+^ T cell activation compared to treatment with single agent MCL1 inhibitor or anti-PD-1. Survival analyses of publicly available data further supported the clinical relevance of this approach, by confirming that higher MCL1 gene expression is associated with lower overall survival for cancer patients treated with anti-PD-1. These results are promising and support testing the combination of MCL1 inhibitors with anti-PD-1, or other ICIs, in advanced melanoma and other cancers that recruit high levels of MDSCs and/or are more sensitive to MCL1 inhibitors themselves.

Based on our previous results, non-sun exposed melanomas, such as uveal and mucosal melanomas, may be good candidates for treatment with MCL1 inhibitors. Uveal and mucosal melanoma cell lines have been shown to be more sensitive to the MCL1 inhibitors than cutaneous melanomas through the inhibitor’s direct killing effects [27, 40]. Therefore, using MCL1 inhibitors in combination with ICIs for these cancers may improve outcomes and kill cancer cells, through the MCL1 inhibitor’s direct killing effects on tumor cells and indirect effects on MDSCs.

This study highlights the importance of investigating how specific BCL2 family members impact immune cells, which may help identify novel therapeutic or prognostic approaches. For example, inhibiting BCL2 with venetoclax has been shown to increase effector T cell frequency and enhance the efficacy of anti-PD-1 [52]. Knockout of BCL2 or inhibiting BCL2 with venetoclax or navitoclax can enhance DC antigen presentation and activation as well as the capacity of DCs to control tumors and to synergize with anti-PD-1 treatment [50]. Additionally, GX15-070, a pan inhibitor of MCL1, BCL2, BCLXL, BCLW, and BFL1, has been shown to induce cell death in Tregs and enhance clearance of lung cancer cells when combined with a rV/F-CEA-TRICOM vaccine [53]. Lastly, for a pro-apoptotic member of the BCL2 family, BIM, high levels of its expression in circulating tumor-reactive T cells were associated with the tumor response to anti–PD-1 therapy [54].

Our scRNA-seq analysis revealed a distinct BCL2 family gene expression pattern in tumor-infiltrating MDSCs from anti-PD-1 resistant tumors. In our analysis, we found that MDSCs coexpressed high levels MCL1 and BCL2A1, but not any of the other populations of tumor-infiltrating myeloid cells captured in our analysis. We have previously shown that overexpressing BCL2A1 in tumor cells make them less susceptible to MCL1 inhibitor [27]. It may be possible to further increase the killing effects on MDSCs by targeting both of these proteins.

Targeting MDSC and their immunosuppressive functions remains an important objective to improve the efficacy of ICIs [5–7, 38]. However, due to their heterogeneous biology and repertoire of immunosuppressive functions [5, 6], a wide variety of approaches are needed to eliminate or inhibit the functions of these cells. Previous preclinical or clinical efforts include inhibiting the trafficking of MDSCs to the tumor microenvironment [5, 6], direct targeting of MDSCs using conventional chemotherapies [5], and differentiating these primarily immature cells into less immunosuppressive subsets using differentiating agents such as ATRA or nutlin-3a [31, 55, 56]. An additional novel therapeutic approach decreases MDSC survival by targeting the unique expression of TRAIL-R2 (TNF-related apoptosis induced ligand-receptor-2) in MDSCs [57]. Here, we show that MCL1 inhibition provides an alternative approach to target MDSCs. Combining these MDSC targeting agents with ICIs has been previously shown to enhance the efficacy of ICIs [56]. Indeed, we found that the combination of the MCL1 inhibitor S64315 and anti-PD-1 decreased melanoma tumor growth further, compared to anti-PD-1 alone. Future experiments combining MCL1 inhibitors with multiple ICIs (anti-PD-1 + anti-CTLA4 or anti-PD-1 + anti-LAG3) may help identify optimal treatment strategies for melanoma, as well as many other tumors where MDSCs prevent the efficacy of conventional therapies.

BH3 mimetics have shown remarkable results in the treatment of hematologic malignancies, but achieving the same level of success in solid tumors has been challenging for many reasons. The lower levels of immune cell infiltration as well as the complex tumor environment has required the use of higher doses of BH3 mimetics in solid tumors. Although there is a great potential to treat melanoma with MCL1 inhibitors in combination with ICIs, the current generation of MCL1 inhibitors are not suitable for use in the clinic due to dose-dependent cardiac toxicity [58]. Cardiomyocytes express high levels of MCL1 and are thus prone to toxic effects from the MCL1 inhibitor. We found that S64315 has a narrow therapeutic window for the tumor inhibitory effect without resulting in off-target toxicity in the mice. To replicate clinical conditions, we treated the mice twice a week with S64315 and found that the highest tolerable dose was 25 mg/kg. To combat drug toxicity, the development of novel formulations and methods for drug delivery of MCL1 inhibitors, specifically to cancer cells and MDSCs, will be beneficial. Furthermore, liver toxicity is one of side effects reported by a fraction of patients treated with ICIs, such as Anti-PD-1 drug Pembrolizumab alone [59–61] or the combination of Anti-CTLA-4 with Anti-PD-1/PD-L1 [62]. Thus, future studies should also evaluate whether the combination of this MCL1 inhibitor with ICIs further aggravates hepatic toxicity.

In summary, this study serves as a “proof-of-concept” for the translation of future BH3 mimetics into clinical trials for melanoma. Using both in vitro and in vivo studies, with human and mouse samples, as well as analyses of a public clinical survival data dataset, this study provides strong evidence for a novel use of the MCL1 inhibitor S64315 to enhance the efficacy of immunotherapies against melanoma. Although the current generation of MCL1 inhibitors are not suitable for use in the clinic, future generations of the compounds may prove to be a powerful new tool for targeting solid tumors and improving patient responses to ICI therapy.

### Reporting summary

Further information on research design is available in the Nature Research Reporting Summary linked to this article.

### Supplementary information

Supplementary Figure 1

Supplementary Figure 2

Supplementary Figure 3

Supplementary Figure 4

Supplementary Table 1

Supplementary Figure legends

Original Western Blot Data

Reporting Summary

## Data Availability

All single-cell RNA-sequencing files are available in GEO (SRA# GSE249909). The code is available at https://github.com/UCHRT/Tobin_Lab.
