# Supplementary figures and images for: MCL1 inhibition targets Myeloid Derived Suppressors Cells, promotes antitumor immunity and enhances the efficacy of immune checkpoint blockade
Source: Cell Death Dis. 2024 Mar 8;15(3):198. doi: 10.1038/s41419-024-06524-w (PMC10923779; doi:10.1038/s41419-024-06524-w)

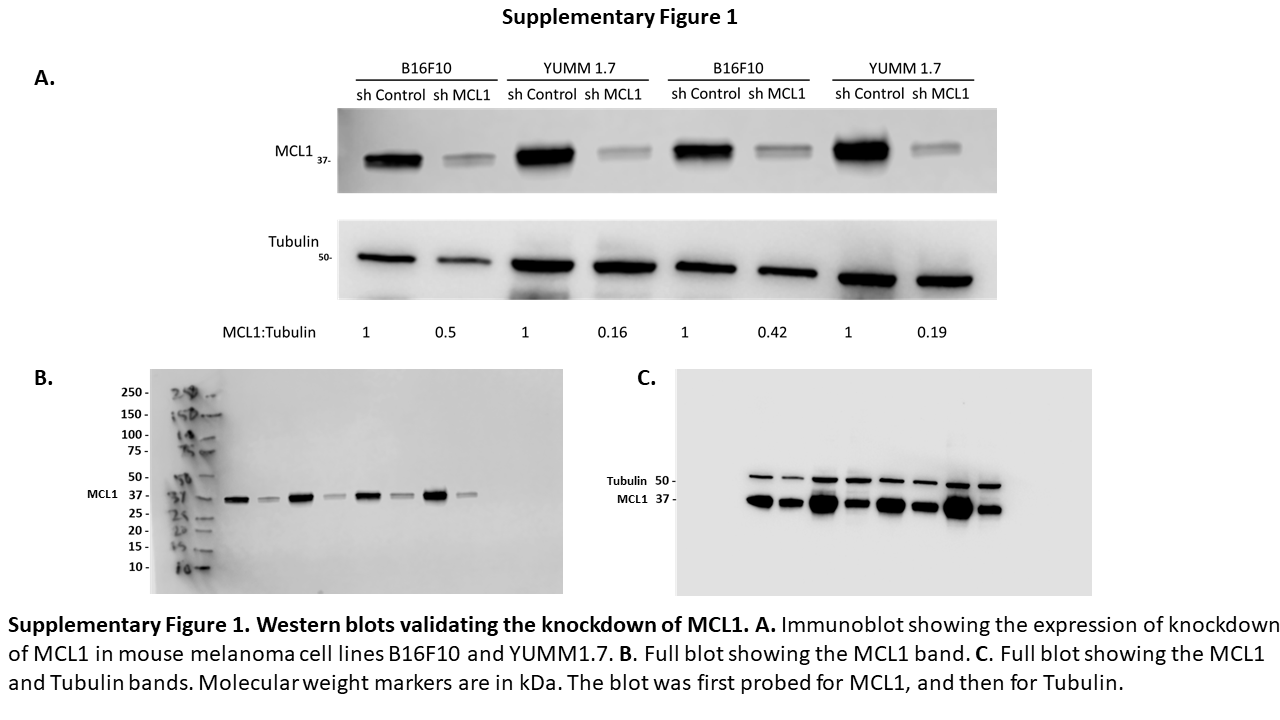

Supplement: Supplementary file 1 — Supplementary Figure 1 [file 41419_2024_6524_MOESM1_ESM.tif]

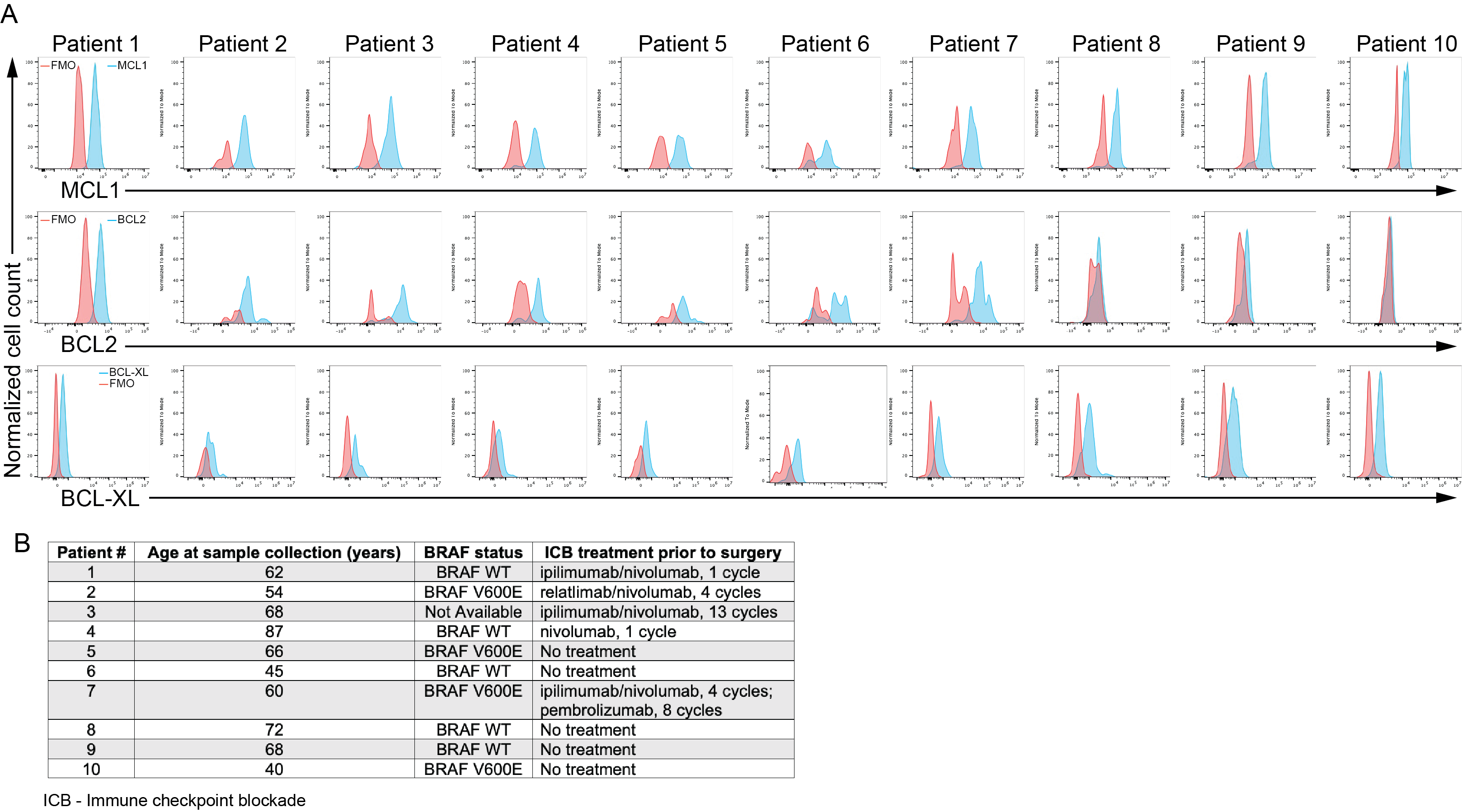

Supplement: Supplementary file 2 — Supplementary Figure 2 [file 41419_2024_6524_MOESM2_ESM.png]

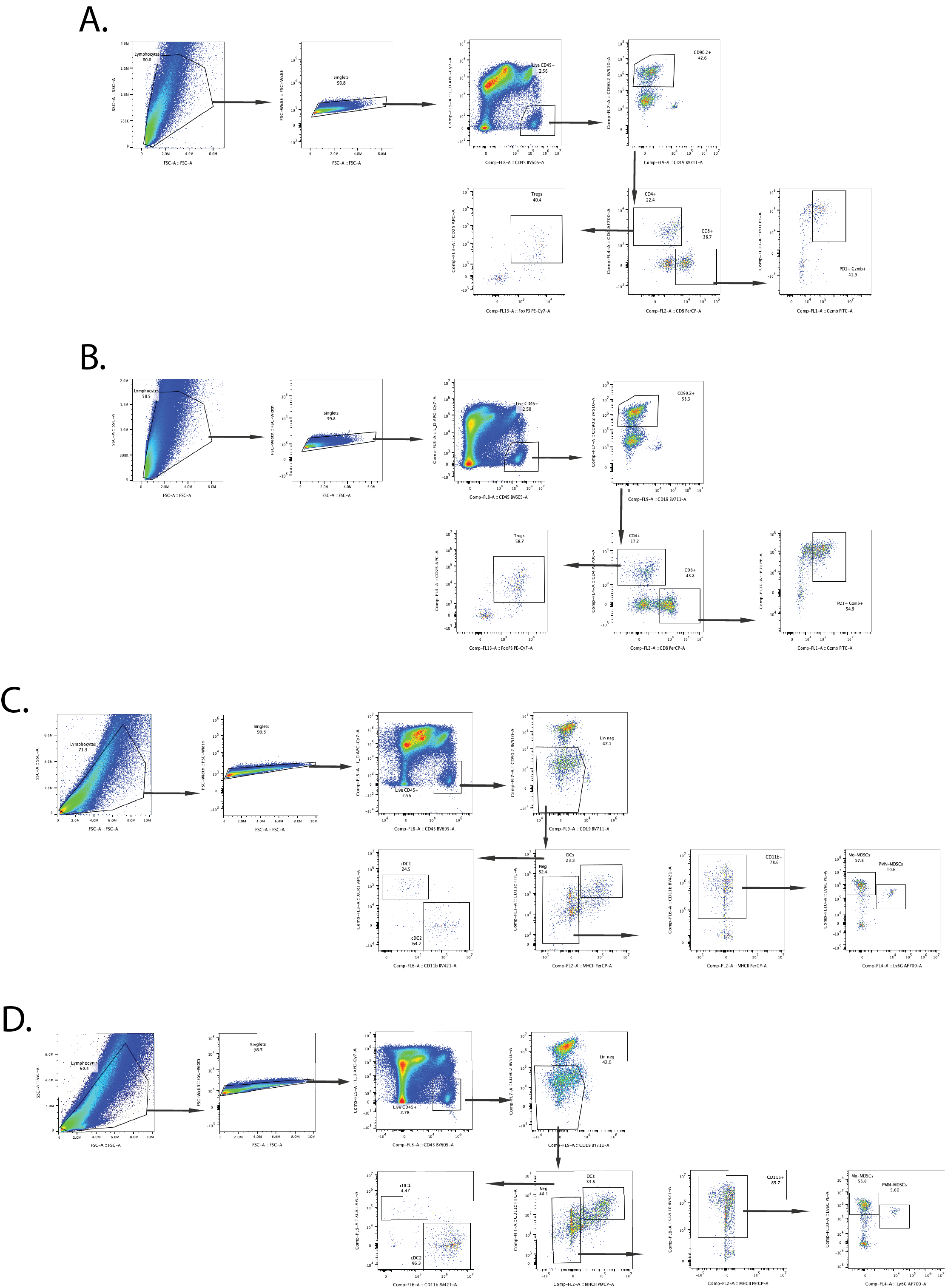

Supplement: Supplementary file 3 — Supplementary Figure 3 [file 41419_2024_6524_MOESM3_ESM.png]

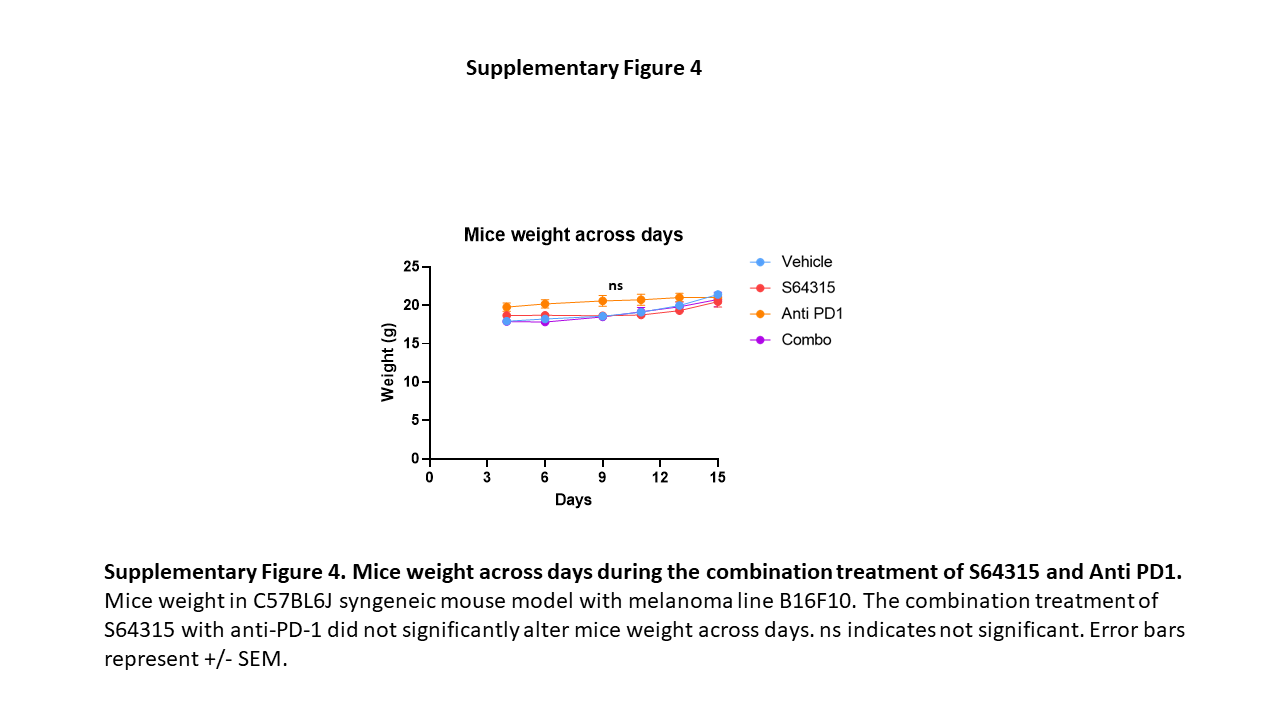

Supplement: Supplementary file 4 — Supplementary Figure 4 [file 41419_2024_6524_MOESM4_ESM.tif]
