# Supplementary material for: MCL1 inhibition targets Myeloid Derived Suppressors Cells, promotes antitumor immunity and enhances the efficacy of immune checkpoint blockade
Source: Cell Death Dis. 2024 Mar 8;15(3):198. doi: 10.1038/s41419-024-06524-w (PMC10923779; doi:10.1038/s41419-024-06524-w)
Supplement: Supplementary file 5 — Supplementary Table 1 [file 41419_2024_6524_MOESM5_ESM.docx]

| Mouse Antibodies | | | | |
| --- | --- | --- | --- | --- |
| **Marker** | **Conjugate** | **Clone** | **Catalog Number** | **Company** |
| Live/Dead | Ghost780 |  | 13-0865-T100 | Cytek |
| CD11b | BV421 | M1/70 | 101235 | BioLegend |
| CD90.2 | BV510 | 30-H12 | 105335 | BioLegend |
| CD45 | BV605 | 30-F11 | 103139 | BioLegend |
| CD19 | BV711 | 6D5 | 115555 | BioLegend |
| PD-1 | PE | 29F.1A12 | 135205 | BioLegend |
| Ly6C | PE | HK1.4 | 128007 | BioLegend |
| CD11c | FITC | N418 | 117305 | BioLegend |
| F480 | PE-Cy7 | BM8 | 123113 | BioLegend |
| FoxP3 | PE-Cy7 | 3G3 | 60-5773-U100 | Cytek |
| Granzyme B | FITC | QA16A02 | 372206 | BioLegend |
| MHCII | PerCP | M5/114.15.2 | 107623 | BioLegend |
| CD8 | PerCP | 53-6.7 | 100731 | BioLegend |
| XCR1 | APC | ZET | 148205 | BioLegend |
| CD25 | APC | PC61 | 102011 | BioLegend |
| Ly6G | Alx700 | 1A8 | 127621 | BioLegend |
| CD4 | Alx700 | GK1.5 | 100430 | BioLegend |

| Human Antibodies | | | | |
| --- | --- | --- | --- | --- |
| **Marker** | **Conjugate** | **Clone** | **Catalog Number** | **Company** |
| Live/Dead | Ghost780 |  | 13-0865-T100 | Cytek |
| CD11b | BV421 | M1/70 | 101235 | BioLegend |
| IFNγ | BV421 | 4S.B3 | 502532 | BioLegend |
| CD45 | BV510 | HI30 | 304035 | BioLegend |
| CD33 | BV605 | P67.6 | 366611 | BioLegend |
| TNF-α | BV605 | MAb11 | 502936 | BioLegend |
| PD-1 | BV605 | EH12.2H7 | 329923 | BioLegend |
| CD14 | BV711 | M5E2 | 301838 | BioLegend |
| Lag3 | PE | 11C3C65 | 369305 | BioLegend |
| BCL2 | PE | BCL/10C4 | 633507 | BioLegend |
| CD15 | PE-Cy7 | W6D3 | 323030 | BioLegend |
| CD8 | PE-Cy7 | SK1 | 344712 | BioLegend |
| Gzmb | FITC | QA16A02 | 372206 | BioLegend |
| MCL1 | Alx488 | Y37 | ab197529 | Abcam |
| CD3 | PerCP | UCHT1 | 300427 | BioLegend |
| CD19 | PerCP | HIB19 | 302227 | BioLegend |
| CD56 | PerCP | 5.1H11 | 362525 | BioLegend |
| TIM3 | APC | F38-2E2 | 345011 | BioLegend |
| BCL-XL | Alx647 | 54H6 | 86387S | CellSignal |
| CD4 | RF710 | OKT4 | 80-00048-T100 | Cytek |
| HLADR | RF710 | L243 | 80-9952-T025 | Cytek |
