# Supplementary material for: MCL1 inhibition targets Myeloid Derived Suppressors Cells, promotes antitumor immunity and enhances the efficacy of immune checkpoint blockade
Source: Cell Death Dis. 2024 Mar 8;15(3):198. doi: 10.1038/s41419-024-06524-w (PMC10923779; doi:10.1038/s41419-024-06524-w)
Supplement: Supplementary file 6 — Supplementary Figure legends [file 41419_2024_6524_MOESM6_ESM.docx]

**Supplementary Figure legends**

**Supplementary Figure 1. Western blots validating the knockdown of MCL1. A.** Immunoblot showing the expression of knockdown of MCL1 in mouse melanoma cell lines B16F10 and YUMM1.7. **B**. Full blot showing the MCL1 band. **C**. Full blot showing the MCL1 and Tubulin bands. Molecular weight markers are in kDa. The blot was first probed for MCL1, and then for Tubulin.

**Supplementary Figure 2. MCL1, BCL2, and BCL-XL expression in human melanoma-infiltrating MDSCs.** **A.** Histograms comparing fluorescence minus one (FMO) staining controls vs. labeled antibody. **B.** Table of clinical data for patients in B.

**Supplementary Figure 3: Gating strategy for mouse tumor studies.** **A.** T cell gating strategy for vehicle control mice. **B.** T cell gating strategy for mice treated with S64315. **C.** Myeloid cell gating strategy for vehicle control mice. **D.** Myeloid gating strategy for mice treated with S64315.

**Supplementary Figure 4. Mice weight across days during the combination treatment of S64315 and Anti PD1.** Mice weight in C57BL6J syngeneic mouse model with melanoma line B16F10. The combination treatment of S64315 with anti-PD-1 did not significantly alter mice weight across days. ns indicates not significant. Error bars represent +/- SEM.
