# Supplementary material for: MCL1 inhibition targets Myeloid Derived Suppressors Cells, promotes antitumor immunity and enhances the efficacy of immune checkpoint blockade
Source: Cell Death Dis. 2024 Mar 8;15(3):198. doi: 10.1038/s41419-024-06524-w (PMC10923779; doi:10.1038/s41419-024-06524-w)
Supplement: Supplementary file 7 — Original Western Blot Data [file 41419_2024_6524_MOESM7_ESM.pdf]

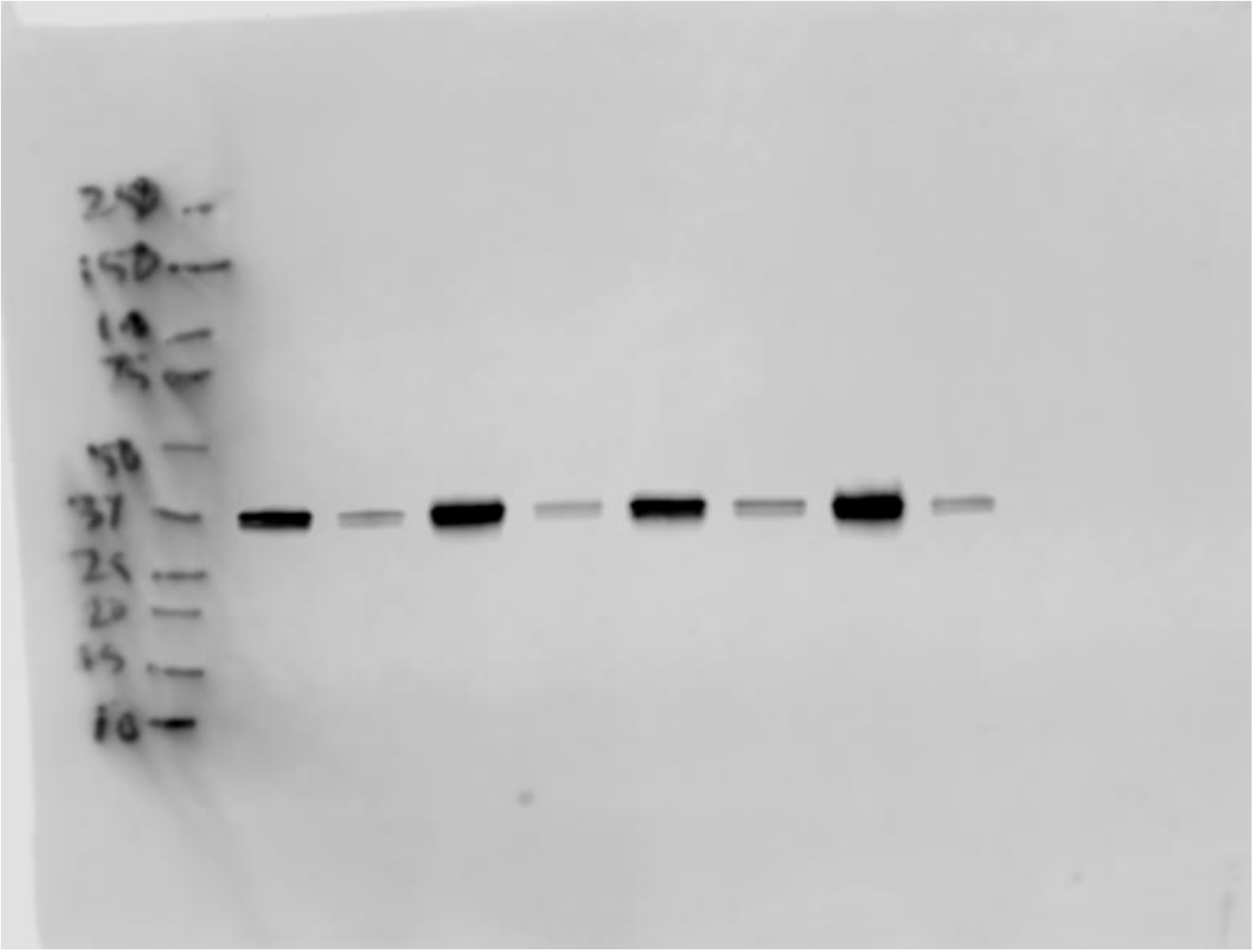

Full blot showing the MCL1 band.

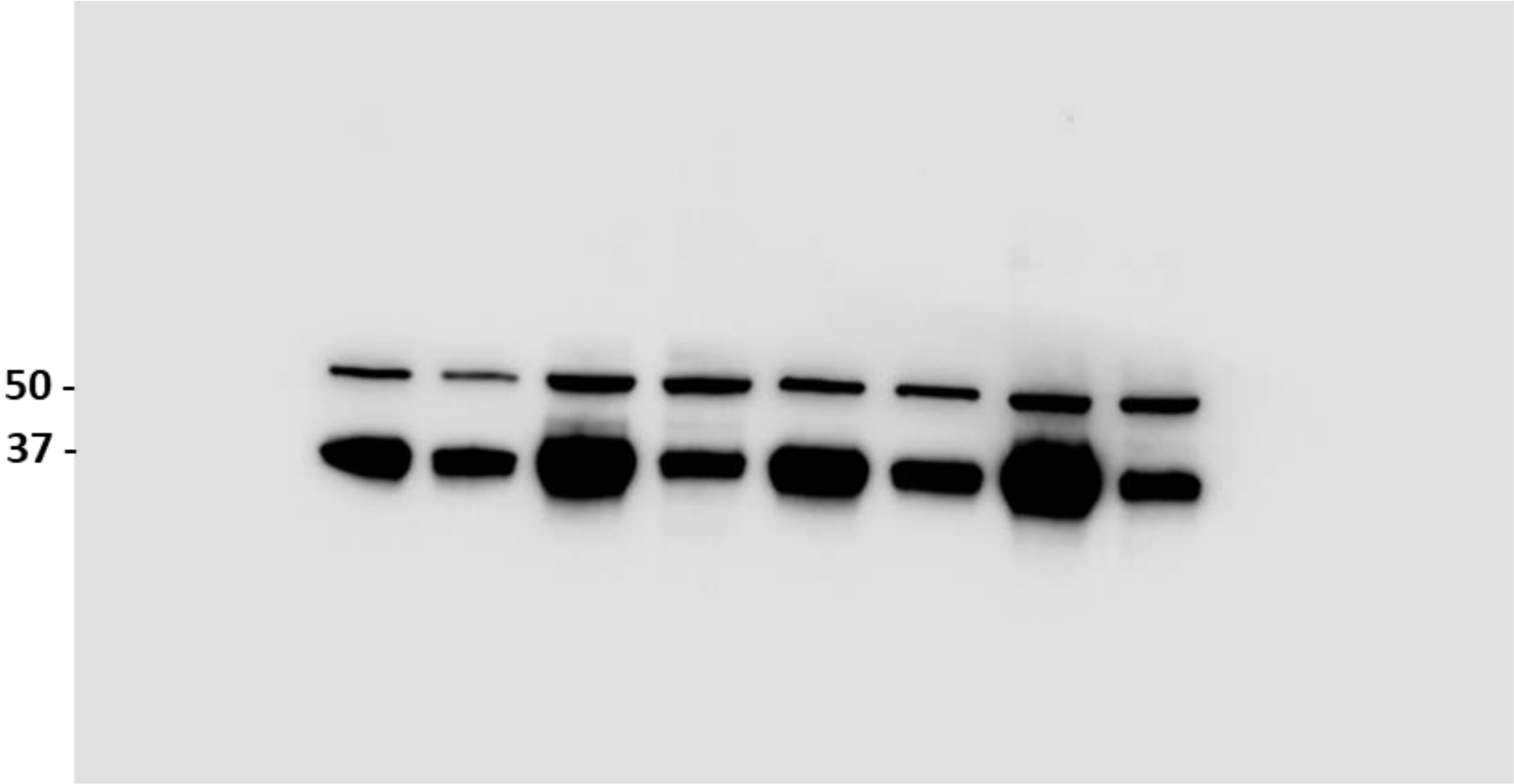

Full blot showing the MCL1 and Tubulin bands. Molecular weight markers are in kDa.
